# Supplementary material for: Fluorescence-Guided Surgery in Pediatric Oncology: Current Practice and Future Directions
Source: Cancers (Basel). 2025 Dec 31;18(1):149. doi: 10.3390/cancers18010149 (PMC12784766; doi:10.3390/cancers18010149)
Supplement: Supplementary file 1 [file cancers-18-00149-s001.zip › cancers-4042924-supplementary.pdf]

Fluorescence-Guided Surgery in Pediatric Oncology: Current Practice and Future Directions

Dominique C. Simons [1,†], Lorenz H. M. van Schalkwijk [1,†], Michiel A. J. van de Sande [1,2], Alexander L. Vahrmeijer [3], Marc H. W. A. Wijnen [1], Alida F. W. van der Steeg [1] and Willemieke S. F. J. Tummers [1,3,*]

1 Princess Maxima Center for Pediatric Oncology, 3584 CS Utrecht, The Netherlands; a.f.w.vandersteeg@prinsesmaximacentrum.nl (A.F.W.v.d.S.)
2 Department of Orthopedic Surgery, Leiden University Medical Center, 2333 ZA Leiden, The Netherlands
3 Department of Surgery, Leiden University Medical Center, 2333 ZA Leiden, The Netherlands
* Correspondence: w.s.f.j.tummers@prinsesmaximacentrum.nl
† These authors contributed equally to this work.

Supplementary Materials

Search string

("Indocyanine Green"[MeSH Terms] OR "fluorescein"[MeSH Terms] OR "fluorescen* guid*"[Title/Abstract] OR "fluorescen* surg*"[Title/Abstract] OR "ICG"[Title/Abstract] OR "indocyanine green"[Title/Abstract] OR "molecular target*"[Title/Abstract] OR "molecular imag*"[Title/Abstract] OR "targeted imag*"[Title/Abstract])

AND

("surgery"[MeSH Subheading] OR "surgical procedures, operative"[MeSH Terms] OR "surg*"[Title/Abstract] OR "operati*"[Title/Abstract] OR "intraoperati*"[Title/Abstract])

AND

("Adolescent"[MeSH Terms] OR "Child"[MeSH Terms] OR "Infant"[MeSH Terms] OR "Pediatrics"[MeSH Terms] OR ("infan*"[Title/Abstract] OR "neonat*"[Title/Abstract] OR "child*"[Title/Abstract] OR "adolescen*"[Title/Abstract] OR "juvenil*"[Title/Abstract] OR "youth*"[Title/Abstract] OR "pediatric*"[Title/Abstract] OR "paediatric*"[Title/Abstract])

AND

("Neoplasms"[Mesh] OR Neoplasm*[TIAB] OR Cancer*[TIAB] OR oncolog*[TIAB] OR tumor*[TIAB] OR tumour*[TIAB] OR Malignan*[TIAB]))
